# Induction chemoimmunotherapy followed by concurrent radiotherapy in patients with locally advanced esophageal cancer: a single-arm phase 2 trial

**DOI:** 10.1093/oncolo/oyaf438

**Published:** 2026-01-06

**Authors:** Hui Chen, Zeyuan Liu, Wang Zheng, Xinchen Sun, Xiaolin Ge, Xiaojie Xia

**Affiliations:** Department of Radiation Oncology, The First Affiliated Hospital with Nanjing Medical University, Nanjing 210029, China; Department of Radiation Oncology, The Affiliated Jiangning Hospital of Nanjing Medical University, Nanjing 210000, China; Department of Oncology, Kangda College of Nanjing Medical University, Haizhou District, Lianyungang 222000, China; Department of Radiation Oncology, The First Affiliated Hospital with Nanjing Medical University, Nanjing 210029, China; Department of Radiation Oncology, The First Affiliated Hospital with Nanjing Medical University, Nanjing 210029, China; Department of Radiation Oncology, The First Affiliated Hospital with Nanjing Medical University, Nanjing 210029, China; Department of Radiation Oncology, The First Affiliated Hospital with Nanjing Medical University, Nanjing 210029, China

**Keywords:** esophageal cancer, immunotherapy, induction therapy, survival, quality of life

## Abstract

**Background:**

This phase II trial prospectively assessed the efficacy and safety of induction chemoimmunotherapy followed by sequential concurrent chemoradiotherapy plus immunotherapy in patients with locally advanced esophageal squamous cell carcinoma (ESCC) who were ineligible for surgery.

**Methods:**

Forty-four patients received 2 cycles of induction therapy (paclitaxel plus carboplatin/nedaplatin combined with a PD-1 inhibitor), followed by concurrent radiotherapy with two additional cycles of chemoimmunotherapy and subsequent immune maintenance therapy for up to 1 year. The primary endpoint was progression-free survival (PFS); the secondary endpoints included overall survival (OS), objective response rate (ORR), disease control rate (DCR), safety, and quality of life (QoL).

**Results:**

At the data cut-off point (median follow-up: 25.5 months), both the ORR and DCR were 95.5%. The median PFS was 26 months (95% CI, 14.8-37.2), and the median OS was 29 months (95% CI, 23.0-35.0). The 1-, 2-, and 3-year PFS rates were 75.0%, 51.9%, and 40.4%, respectively, and the OS rates were 81.8%, 63.1%, and 42.0%, respectively. Distant metastasis represented the main failure mode (64.0%). Treatment-related adverse events were generally mild; moreover, 17 patients (38.6%) experienced grade ≥3 events, primarily involving hematologic toxicity (14/17). Severe immune-related adverse events were rarely observed. QoL assessment in surviving patients (*n* = 21) indicated favorable overall function and well-being.

**Conclusions:**

This regimen of induction chemoimmunotherapy followed by concurrent chemoradiotherapy and maintenance immunotherapy demonstrated promising survival outcomes, a manageable safety profile, and a preserved QoL, thereby offering a viable nonsurgical alternative for patients with locally advanced ESCC.

Implications for Practice This study demonstrated that induction chemoimmunotherapy followed by concurrent chemoradiotherapy and maintenance immunotherapy provides promising survival outcomes with a manageable safety profile for patients with locally advanced esophageal squamous cell carcinoma who are ineligible for surgery. This regimen, which is associated with high response rates and prolonged survival while preserving quality of life, offers a viable nonsurgical treatment alternative. These findings support the integration of this multimodal approach into clinical practice, thus potentially changing the standard of care for this patient population by improving long-term disease control and tolerability.

## Introduction

Esophageal cancer ranks seventh throughout the world in terms of cancer-related deaths. A study focusing on the distribution of esophageal cancer in Asia revealed that the incidence rate in China is approximately 224 000 cases per 382 000 individuals,^1^^,^^2^ thus demonstrating this cancer as the fifth-highest cause of cancer-related mortality in the country. The lack of typical clinical manifestations in the early stages results in more than two-thirds of patients being diagnosed with metastatic or locally advanced disease, which poses significant challenges for early detection and treatment.^3^

The RTOG-8501 trial solidified the use of concurrent chemoradiotherapy as the established standard for treating locally advanced, unresectable esophageal cancer.^4^ Recent investigations have delved into diverse approaches to increase survival rates in this patient population, such as exploring different chemotherapy regimens,^5^^,^^6^ increasing radiotherapy doses,^7^ and incorporating targeted therapies^8^ and immunotherapies.^9^ Despite these achievements, the 5-year survival rate for patients with esophageal cancer in China has remained below 30%, thereby underscoring the ongoing need to optimize current treatment modalities.

The advent of immune checkpoint blockade therapy has significantly transformed the management of esophageal cancer. The current standard of care for advanced esophageal squamous cell carcinoma (ESCC) involves the integration of immunotherapy with chemotherapy as a first-line treatment.^10^ Recent studies have explored the role of immunotherapy in the treatment of locally advanced ESCC, with small-scale studies suggesting that the combination of chemoradiotherapy and immunotherapy yields promising safety and efficacy outcomes in patients with unresectable esophageal cancer.^11^^,^^12^ Ongoing phase III trials, such as the KEYNOTE-975 (NCT04210115) and RATIONALE-311 (NCT03957590) trials, are further evaluating this therapeutic strategy. Chemotherapy increases the immunogenicity of tumor cells, whereas radiotherapy facilitates the release of tumor antigens, thereby eliciting an immune response that may enhance the efficacy of immunotherapy.^13^^,^^14^ The utilization of induction chemotherapy in conjunction with immunotherapy not only enables the early control of micrometastases but also leads to primary tumor reduction, thereby establishing an optimal window for subsequent radiotherapy.^15^

A gap in the literature exists regarding the combined use of induction immunochemotherapy followed by concurrent radiotherapy in the context of locally advanced esophageal cancer. Therefore, we designed this phase II clinical trial to assess the efficacy and safety of induction chemotherapy and immunotherapy followed by concurrent radiotherapy in patients with locally advanced esophageal cancer. Through this investigation, we aimed to explore a potentially more effective and viable therapeutic regimen, thus offering new treatment options for this patient population.

## Methods

### Study design and patients

This single-arm phase II clinical trial is prospective in nature and focuses on patients diagnosed with locally advanced ESCC. The trial, which was conducted at the First Affiliated Hospital of Nanjing Medical University between January 2022 and June 2023, involved the administration of induction chemoimmunotherapy followed by concurrent radiotherapy. The inclusion criterion included patients aged 18 years and older with histopathologically or cytologically confirmed stage II-IVB ESCC according to the American Joint Committee on Cancer’s eighth edition staging system (2018). Stage IVB disease is limited to nonregional lymph node metastasis and is devoid of distant organ metastasis. Patients were treatment-naive, possessed adequate organ function, and demonstrated an Eastern Cooperative Oncology Group performance status score of 0-1. The exclusion criterion was a history of malignancy, autoimmune disorders, or severe comorbidities. This study was conducted in accordance with the ethical principles outlined in the Declaration of Helsinki. The protocol was approved by the Medical Ethics Review Committee of the First Affiliated Hospital of Nanjing Medical University. This trial is registered with ClinicalTrials.gov (NCT07015489).

### Histopathologic grading

The histopathologic grading of the tumors was performed according to the World Health Organization (WHO) Classification of Tumors (2019). Tumors were classified into three grades: Grade 1 (well differentiated), Grade 2 (moderately differentiated), and Grade 3 (poorly differentiated). Tumor differentiation was determined by pathologists via histopathologic examination of tumor samples.

### Treatment

All of the patients underwent 2 cycles of induction chemoimmunotherapy, followed by 2 cycles of concurrent chemoimmunotherapy during radiotherapy. Patients were subsequently maintained on immunotherapy for 1 year after radiotherapy until the occurrence of disease progression (PD) or intolerance.

The chemotherapy regimens consisted of paclitaxel (135 mg/m^2^ on Day 1), carboplatin (AUC = 5 on Day 2) or nedaplatin (75 mg/m^2^ on Day 2), which were administered every 3 weeks. The carboplatin infusion occurred for 30 minutes and was accompanied by standard antiemetic treatment. Furthermore, patients received an intravenous treatment of programmed death-1 (PD-1) monoclonal antibody every 3 weeks until the occurrence of disease progression or intolerable adverse effects for a maximum of one year. The PD-1 inhibitors that were primarily used in this study included camrelizumab and tislelizumab, although other PD-1 inhibitors were permitted based on patient-specific considerations.

The intensity-modulated radiotherapy (IMRT) technique was used to irradiate the involved areas. The cervical and upper thoracic lesions required irradiation of the bilateral supraclavicular areas and the high-risk lymphatic drainage areas in the upper mediastinum. The delineation of the gross tumor volume (GTV) for the esophageal tumor was meticulously outlined layer by layer on the enhanced CT images by incorporating information from barium esophageal meal, gastroscopy, ultrasonic gastroscopy, or PET-CT scans. The clinical target volume (CTV) extended outwards axially from the GTV by 0.6-0.8 cm, as well as superiorly and inferiorly by 2-3 cm. The planning target volume (PTV) was defined as a 0.5-0.8 cm expansion of the CTV. Metastatic lymph nodes were demarcated as the gross tumor volume of nodes (GTVnd), with the planning target volume for nodes (PTVnd) being generated by expanding the GTVnd by 0.8-1 cm.

The dose requirements for 95% of the PTV and PTVnd were 50-50.4 Gy delivered in 25-28 fractions, with a per-fraction dose ranging from 1.8 to 2.0 Gy. The dose distribution within the target volume and exposure to organs at risk were assessed slice-by-slice in the transverse plane. This assessment was subsequently integrated with dose-volume histogram (DVH) analysis to determine the optimal treatment plan. The treatment protocol included a maximum spinal cord dose of <45 Gy, a lung volume dose of ≥20 Gy (V20) <28%, a lung volume dose of ≥30 Gy (V30) <20%, and a mean lung dose of ≤15 Gy.

### Assessment of treatment response

Treatment response was assessed using a combination of imaging and endoscopic biopsy. Within 1 month after radiotherapy, all patients underwent barium esophagography, esophageal MRI, and cervicothoracic CT to evaluate tumor response. Endoscopic biopsy was performed within 1–3 months post–radiotherapy to assess pathological response. Although subject to false negatives, biopsy served as a supplementary tool alongside imaging to improve evaluation accuracy.

Follow-up was conducted every 3 months for the first 2 years post–treatment, then every 6 months thereafter. Evaluations included imaging (CT/MRI), endoscopy, tumor marker tests, and clinical symptom assessment.

### Endpoints

All of the patients underwent follow-up assessments beginning on Day 28 after the completion of treatment, with evaluations being conducted every 3 months during the initial 2 years and every 6 months thereafter. The primary endpoint was progression-free survival (PFS), which was defined as the duration from treatment initiation to either tumor progression or all-cause mortality. The secondary endpoints included overall survival (OS, which was defined as the interval between treatment initiation and death from any cause), the objective response rate (ORR, which included the proportions of patients with complete response [CR] and partial response [PR]), the disease control rate (DCR, which involved the proportions of patients with CR, PR, or stable disease [SD]), and safety and treatment-related toxicity. The therapeutic response was evaluated in accordance with the response evaluation criteria for solid tumors (RECIST) version 1.1.^16^ The assessment of safety and toxicity adhered to the National Cancer Institute Common Terminology Criteria for Adverse Events (CTCAE) version 5.0.

### Quality of life evaluation

All of the surviving patients were surveyed via a quality of life (QoL) questionnaire that included assessments of physical well-being, familial support, emotional and functional status, and swallowing capacity. The questionnaire was constructed by integrating elements from the functional assessment of cancer therapy-general (FACT-G) and functional assessment of cancer therapy-esophageal (FACT-E) scales. The QoL questionnaire was completed during the final follow-up in June 2025, which represented the last follow-up visit of the study. This assessment provided insights into the long-term effects of treatment and patients’ overall well-being after the completion of therapy.

### Statistical analyses

This single-arm, prospective phase II clinical study was designed with sample size calculations based on the primary endpoint of investigator-assessed 2-year PFS. A previous study suggested that the combination of immunotherapy with concurrent radiotherapy in patients with local ESCC yielded a 2-year PFS rate of 65.0%.^17^ This study aimed to increase this outcome to 75%. Sample size calculations were performed via the log-rank test method in NCSS PASS 2021 software, with parameters set at *ɑ* = 0.05, power = 0.8, P0 = 65%, and P1 = 75%. The analysis indicated that 39 participants were required for enrolment. After factoring a 10% dropout rate, a total of 44 participants were ultimately required for the study.

Data analysis was conducted with SPSS software version 26. Descriptive statistics were used to summarize the demographic characteristics, baseline characteristics, efficacy indicators, and safety data. Continuous variables are reported as the means ± standard deviations, and categorical variables are reported as frequencies and percentages. Student’s *t*-tests were used for assessing the quantitative variables, whereas chi-square tests or Fisher’s exact tests were utilized for qualitative variables. OS and PFS were estimated via the Kaplan–Meier method. During the preparation of this manuscript, the author used DeepSeek software to improve the language.

## Results

### Patients

Forty-four patients diagnosed with locally advanced ESCC were included in this study. The cohort predominantly consisted of male individuals (86.4%), and 63.6% were aged 65 years or older. Most of the patients demonstrated a good performance status, as indicated by 68.2% of the patients exhibiting an ECOG score of 0. Notably, 47.7% and 43.2% of the patients reported histories of smoking and alcohol consumption, respectively. T3 tumors were the most prevalent tumors (72.7%), and N1 nodal status was observed in 61.4% of the cases. A total of 18.2% of the patients were classified as M1, all of whom presented with nonregional lymph node metastases only. Stage III disease was predominant and accounted for 47.7% of the cases. The tumors were primarily located in the middle of the thoracic esophagus or involved overlapping segments. The majority of the participants received a semiliquid diet (68.2%). Moreover, the tumor size was observed to be ≥5 cm in 65.9% of the patients. The detailed baseline clinical characteristics of the study cohort are presented in Table 1.

**Table 1.** Baseline characteristics of patients.

Characteristic	*N* = 44
**Age**	
**<65**	16 (36.4%)
**≥65**	28 (63.6%)
**Gender**	
**Female**	6 (13.6%)
**Male**	38 (86.4%)
**BMI**	
**<18.5**	6 (13.6%)
**18.5-24**	23 (52.3%)
**≥24**	15 (34.1%)
**Smoking**	
**Yes**	21 (47.7%)
**No**	23 (52.3%)
**Alcohol abuse**	
**Yes**	19 (43.2%)
**No**	25 (56.8%)
**ECOG score**	
**0**	30 (68.2%)
**1**	14 (31.8%)
**T**	
**T2**	8 (18.2%)
**T3**	32 (72.7%)
**T4**	4 (9.1%)
**N**	
**N0**	6 (13.6%)
**N1**	27 (61.4%)
**N2**	10 (22.7%)
**N3**	1 (2.3%)
**M**	
**M0**	36 (81.8%)
**M1**	8 (18.2%)
**Stage**	
**II**	11 (25.0%)
**III**	21 (47.7%)
**IV**	12 (27.3%)
**Tumor location**	
**Cervical**	2 (4.6%)
**Upper chest**	9 (20.4%)
**Middle chest**	11 (25.0%)
**Lower chest**	6 (13.6%)
**Overlapping**	16 (36.4%)
**Grade**	
**G1**	6 (13.6%)
**G2**	21 (47.7%)
**G3**	17 (38.6%)
**Tumor size**	
**<5 cm**	15 (34.1%)
**≥5 cm**	29 (65.9%)
**Nutritional management mode**	
**Soft diet**	7 (15.9%)
**Semi-liquid diet**	30 (68.2%)
**Liquid diet**	7 (15.9%)

Abbreviation: BMI, body mass index.

### Efficacy

All of the patients underwent assessment for treatment efficacy. CR was observed in 30 patients, PR in 12 patients, and progressive disease (PD) in 2 patients. Both the ORR and the DCR were determined to be 95.5% (Table 2).

**Table 2.** Short-term efficacy of patients.

Event	Patient (*N* = 44)
**CR**	30 (68.2%)
**PR**	12 (27.3%)
**SD**	0 (0%)
**PD**	2 (4.5%)
**ORR**	42 (95.5%)
**DCR**	42 (95.5%)

Abbreviations: CR, complete response; DCR, disease control rate; ORR, objective response rate; PD, progressive disease; PR, partial response; SD, stable disease.

**Figure 1.**
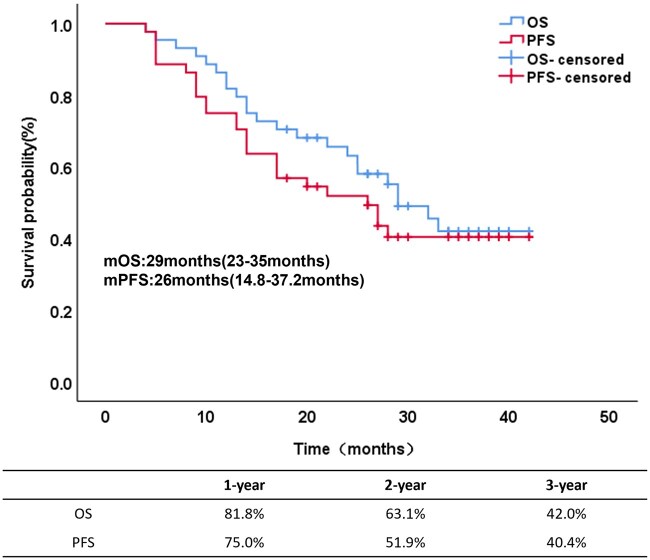
Kaplan–Meier curves of overall survival and progression-free survival. Abbreviations: mOS, median overall survival; mPFS, median progression-free survival; OS, overall survival; PFS, progression-free survival.

Until June 2025, patients were followed up for a median duration of 35 months (range: 29-40 months). Analysis via the Kaplan–Meier method revealed a median PFS (mPFS) of 26 months (95% confidence interval [CI], 14.8-37.2 months) and a median OS (mOS) of 29 months (95% CI, 23.0-35.0 months). The PFS rates at 1, 2, and 3 years were 75%, 51.9%, and 40.4%, respectively, with corresponding OS rates of 81.8%, 63.1%, and 42%, respectively, as depicted in Figure 1. Among the 44 patients, 25 (56.8%) experienced treatment failure events during the follow-up period, whereas 19 (43.2%) did not experience disease progression at the last follow-up. The most common site of first failure was distant metastasis (12 patients, 27.3%). Localized regional recurrence was noted in 6 patients (13.6%), and local combined distant recurrence was detected in 4 patients (9.1%). Additionally, 1 patient (2.3%) developed a secondary primary cancer, and 2 patients (4.6%) experienced sudden death from unknown causes (Table 3).

**Table 3.** Patterns of treatment failure.

Type of event	Patient (*N* = 44)
**Live without treatment failure**	19 (43.2%)
**Failure**	25 (56.8%)
**Locoregional only**	6 (13.6%)
**Distant only**	12 (27.3%)
**Locoregional and distant**	4 (9.1%)
**Second primary cancer**	1 (2.3%)
**Sudden death for unknown reasons**	2 (4.6%)

We performed exploratory analyses to assess outcomes in key patient subgroups. Patients who achieved a CR after induction exhibited significantly better survival than those who achieved a PR, with 1-year OS rates of 93.3% versus 58.3% and 1-year PFS rates of 90.0% versus 41.7% (both *P* < 0.05). A trend toward improved survival was observed in patients with a PD-L1 CPS ≥1 than those with a CPS <1, which was statistically significant for PFS (1-year PFS: 72.4% vs 58.3%) but not for OS (1-year OS: 82.8% vs 57.1%). No statistically significant differences in OS or PFS were observed between patients with grade 1-2 lymphopenia and those with grade 3-4 lymphopenia (all *P* > 0.05).

### Safety

A total of 44 patients were assessed for treatment-related toxicities in this study (Table 4), which were graded according to the CTCAE 5.0 criteria. Hematologic toxicity was notable, with leukopenia (≥ grade 3 in 14 cases, 31.8%), thrombocytopenia (≥ grade 3 in 4 cases, 9.1%), and anemia (no grade ≥3) being commonly reported. Mild hepatic and renal impairments were observed, which predominantly presented as grade 1 transaminase elevations, with only 1 case being graded as 4. Minimal renal abnormalities were detected, with only 2 cases being graded as 1, and no cases ≥ grade 2 being observed. Gastrointestinal toxicities primarily included stomatitis, anorexia, vomiting, belching, diarrhea, and constipation, all of which did not have severe manifestations (no grade ≥3).

**Table 4.** Treatment-related adverse events.

Event	*N* = 44				
	0	1	2	3	4
**Leukocytopenia**	5	7	18	10	4
**Thrombocytopenia**	16	13	11	1	3
**Hemoglobin decrease**	6	21	17	0	0
**Transaminase elevation**	29	11	2	1	1
**Renal dysfunction**	42	2	0	0	0
**Stomatitis**	33	8	3	0	0
**Dysphagia**	10	27	5	2	0
**Fatigue**	25	14	5	0	0
**Anorexia**	10	27	6	1	0
**Vomiting**	26	16	2	0	0
**Hiccups**	29	14	1	0	0
**Diarrhea**	37	4	3	0	0
**Constipation**	26	18	0	0	0
**Cough**	28	15	1	0	0
**Fever**	40	4	0	0	0
**RE**	17	13	11	2	1
**RP**	33	8	3	0	0
**RD**	32	10	0	2	0
**irAEs**	31	8	2	2	1

Abbreviations: irAEs, immune-related adverse events; RD, radiation dermatitis, RE, radiation esophagitis; RP, radiation pneumonitis.

Radiation-induced adverse effects included grade 3 and 4 esophagitis in 4.5% and 2.3% of the patients, respectively, along with grade 3 dermatitis being reported in 4.5% of the patients. Radiation pneumonitis was mostly reported to be mild, with no cases of grade ≥3 being observed. Immune-related toxicities consisted of grade 3 events (4.5%) and grade 4 events (2.3%), with the remainder of the toxicities being defined as grades 1-2. A total of 13 patients (31%) experienced immune-related adverse events (irAEs), including 2 patients with grade 3 immune-related dermatitis and hepatitis, as well as 1 patient with grade 4 immune-related hepatitis. No severe (≥ grade 3) immune-related pneumonitis was observed. These irAEs were promptly managed with appropriate interventions and immunosuppressive therapies.

Overall, 17 patients (38.6%) experienced grade ≥3 adverse events, the majority (14/17) of which were hematologic toxicities. Treatment-related toxic side effects predominantly involved grade 1-2 effects, and the toxicity profile remained manageable, with no treatment interruptions or fatalities being attributed to toxicity.

### Quality of life

At the final follow-up, 21 surviving patients participated in a QoL questionnaire assessment based on the integration of the FACT-G and FACT-E scales. The findings revealed positive physical well-being among the patients, with 85.7% reporting no weakness, 95.2% reporting no pain, and 85.7% not being troubled by treatment side effects (Table 5).

**Table 5.** Quality of life assessment in patients.

Issue	No.	Item	Not at all, No. (%)	A little bit, No. (%)	Somewhat, No. (%)	Quite a bit, No. (%)	Very much, No. (%)
**Physical well-being**	1	I have a lack of energy.	18 (85.7)	3 (14.3)	0	0	0
	2	I have pain.	20 (95.2)	1 (4.8)	0	0	0
	3	I am bothered by the side effects of treatment.	18 (85.7)	3 (14.3)	0	0	0
**Family well-being**	4	I get emotional support from my family.	0	0	1 (4.8)	2 (9.5)	18 (85.7)
	5	My family has accepted my illness.	0	0	0	2 (9.5)	19 (90.5)
	6	Family communication about my illness is poor.	0	0	0	2 (9.5)	19 (90.5)
**Emotional well-being**	7	I am losing hope in the fight against my illness.	19 (90.5)	2 (9.5)	0	0	0
	8	I feel nervous.	18 (85.7)	2 (9.5)	1 (4.8)	0	0
	9	I worry about dying.	17 (81.0)	2 (9.5)	1 (4.8)	1 (4.8)	
**Functional well-being**	10	I have been able to work (including housework).	0	2 (9.5)	1 (4.8)	2 (9.5)	16 (76.2)
	11	I am able to enjoy life “in the moment”.	0	0	1 (4.8)	1 (4.8)	19 (90.5)
	12	I am content with the quality of my life right now.	0	1 (4.8)	0	2 (9.5)	18 (85.7)
**Swallowing and eating**	13	I am able to eat the foods that I like.	0	0	1 (4.8)	2 (9.5)	18 (85.7)
	14	I can swallow naturally and easily.	0	0	1 (4.8)	2 (9.5)	18 (85.7)
	15	I am losing weight.	18 (85.7)	3 (14.3)	0	0	0

In the domain of family support, more than 90% of the patients perceived their families as being accepting of their illness, with 85.7% reporting high levels of emotional support and effective communication within the family. With respect to emotional well-being, 90.5% of the patients remained optimistic about their cancer treatment; moreover, 85.7% demonstrated no signs of anxiety, and 81.0% expressed no worries about mortality. With respect to functional capacity, 76.2% of the patients indicated the ability to perform their routine activities, including household tasks. Additionally, 90.5% of the patients reported a positive life perspective, and 85.7% were content with their current QoL. In terms of swallowing and food intake, 85.7% of the patients demonstrated normal functionality. The efficacy of weight maintenance was observed to be satisfactory, with 85.7% of the participants not experiencing significant weight loss. A comprehensive assessment of the patient cohort revealed that those patients receiving a combination of induction chemoimmunotherapy and radiotherapy exhibited optimal physical function, emotional health, and social support, as well as improved QoL throughout the extended follow-up period.

## Discussion

The rapid progress in immunotherapy for treating esophageal cancer has led to the widespread recommendation of immune checkpoint inhibitors (ICIs) as the primary treatment for patients with advanced disease. However, in individuals with locally advanced, unresectable ESCC, the current standard treatment is indicated as definitive concurrent chemoradiotherapy (dCCRT), which is associated with a dismal prognosis, with an mOS of merely 9-10 months being reported.^18^ The synergistic interplay between radiotherapy and immunotherapy has garnered increasing attention in recent years because of its well-established biological rationale. Nonetheless, the optimal timing and strategy for incorporating immunotherapy into dCCRT are still subjects of ongoing investigation.

This study provides the first systematic evaluation of a comprehensive regimen—induction chemoimmunotherapy followed by concurrent chemoradiotherapy plus immunotherapy—for locally advanced unresectable ESCC. The protocol demonstrated promising efficacy, with an ORR of 95.5%, mPFS of 26 months, and mOS of 29 months. Severe (grade ≥3) treatment-related adverse events were uncommon, indicating favorable tolerability. Throughout follow-up, most patients sustained satisfactory QoL across physical, emotional, social, and swallowing domains, suggesting that this approach offers both survival benefit and preserved QoL.

Compared with similar investigational strategies that integrated immunotherapy with definitive radiotherapy in thoracic oncology (such as the PACIFIC-2 trial^19^ [utilizing concurrent durvalumab with chemoradiation for non–small cell lung cancer] or the JAVELIN Lung 100 trial^20^ [utilizing maintenance avelumab postchemoradiation for non–small cell lung cancer], which did not demonstrate significant survival improvements), the positive outcomes observed in our study may be attributed to several pivotal differences. First, ESCC and NSCLC possess fundamentally distinct tumor biological characteristics and immune microenvironments, which may lead to different responses to immunomodulatory strategies. Second, the treatment strategies significantly differed: our study utilized sequential induction chemoimmunotherapy followed by concurrent chemoradiation, aiming to activate immunity and reduce tumor burden, unlike the purely concurrent or maintenance models used in the referenced lung cancer trials. Third, the patient populations varied: our trial focused on locally advanced ESCC within a neoadjuvant setting, whereas the NSCLC trials involved unresectable stage III disease, which often includes bulky T4 lesions and a potentially greater tumor burden. Finally, a key technical distinction lies in radiotherapy technique: our use of modern involved-field irradiation resulted in smaller target volumes and less exposure to lymphoid organs compared to the larger fields required for bulky T4 NSCLC, likely contributing to enhanced treatment tolerance and efficacy by better preserving systemic immune function.

Mechanistically, ICIs function by reversing T-cell exhaustion and restoring antitumor immune responses. In addition to its direct cytotoxic effects, radiotherapy can enhance host immunity through various mechanisms, including reshaping the tumor immune microenvironment and augmenting T-cell infiltration^21^; inducing rapid tumor cell death and the release of neoantigens and proinflammatory mediators to stimulate antigen presentation and immune activation^22^; increasing PD-L1 expression to modulate T-cell function^14^; and increasing MHC-I expression to heighten tumor antigen exposure to cytotoxic T lymphocytes (CTLs).^14^^,^^22^ Notably, the cGAS-STING pathway plays a pivotal role in radiotherapy-induced immune activation.^23^ The PD-L1 expression orchestrated by this pathway can be counteracted by ICI blockade, thus aiding in overcoming immune evasion and increasing antitumor efficacy.

Resistance to radiotherapy is frequently linked to tumor hypoxia, which arises from aberrant or impaired tumor vasculature. Hypoxic environments diminish DNA damage induced by ionizing radiation^24^ and enhance radioresistance through the stabilization of hypoxia-inducible factor-1α (HIF-1α).^25^ Recent research has indicated that ICIs not only stimulate T cells but also normalize the tumor vasculature,^26^^,^^27^ thereby ameliorating hypoxia and potentially enhancing tumor sensitivity to radiation. This mechanism demonstrates promise for the synergistic effects of combining radiotherapy with immunotherapy.

This mechanistic synergy is also supported by clinical evidence. The EC-CRT-001 trial (which was the first prospective study to explore concurrent PD-1 blockade with CRT) achieved a 1-year OS rate of 78.4%, which was notably higher than the 56%-70% rate reported in historical CRT studies.^9^ In addition, a multicentre real-world study presented at the 2024 ASTRO Annual Meeting demonstrated that compared with CRT alone, consolidation immunotherapy following definitive CRT significantly prolonged PFS and OS in patients with locally advanced ESCC. After propensity score matching, the immunotherapy group achieved a median OS of 27.4 months versus the OS of 9.3 months observed in the CRT-alone group (HR = 0.47; *P* = 0.0009).^28^ A multicentre phase II trial enrolling 75 patients with locally advanced ESCC reported that induction sintilimab plus chemotherapy followed by CRT yielded a 2-year local control rate of 81.7% (95% CI, 72.7%-90.7%), thus significantly exceeding the rate of 71.3% reported in the historical ESO-Shanghai2 cohort treated with CRT alone.^27^ These findings further strengthen the clinical potential of integrating PD-1 blockade into standard CRT regimens to improve outcomes in patients with inoperable locally advanced ESCC while underscoring the need for validation in large-scale randomized controlled trials.

In the neoadjuvant setting, the CROSS and NEOCRTEC501 trials established neoadjuvant chemoradiotherapy (nCRT) followed by surgery as a standard approach for treating resectable esophageal cancer.^29^^,^^30^ However, the JCOG1109 trial demonstrated that compared with standard doublet chemotherapy, triplet chemotherapy significantly improved survival, whereas the addition of radiotherapy in the preoperative regimen did not confer further survival benefits.^31^ These findings suggest that the advantage of combining radiotherapy with chemotherapy may be context dependent, thus underscoring the importance of optimizing patient selection. Recent evidence indicates that the earlier integration of immunotherapy into the neoadjuvant phase may also yield substantial benefits. A randomized phase III ESCORT-NEO trial revealed that ICI-based regimens significantly increased pathological CR (pCR) rates and improved postoperative survival outcomes in patients with ESCC without compromising safety. Furthermore, a large real-world cohort study conducted by Professor Zhigang Li’s team reinforced the superiority of neoadjuvant chemoimmunotherapy over nCRT, reporting higher 2-year OS rates (81.3% vs 71.3%), longer disease-free survival (DFS), and a lower incidence of distant metastasis (13.5% vs 25.0%).^32^^,^^33^ Collectively, these findings indicate that immunotherapy has the potential to enhance systemic disease control and (when appropriately timed and integrated) may complement or even surpass the therapeutic benefits of radiotherapy.

Building upon this work, our research presents a novel three-phase treatment protocol comprising “induction chemoimmunotherapy → concurrent chemoradiotherapy + immunotherapy → maintenance immunotherapy.” Notably, the study cohort predominantly consisted of patients with advanced-stage disease (75.0% stage III–IV), substantial tumor size (65.9% with a tumor diameter ≥5 cm), and M1 stage (confined to nonregional lymph node metastasis) disease. Nevertheless, the regimen demonstrated encouraging efficacy, thus suggesting its potential suitability even for high-risk patient populations.

In terms of safety, the majority of the adverse events were classified as grades 1-2, with grade ≥3 toxicities being rarely observed and no treatment-related fatalities being recorded. Longitudinal assessments of QoL revealed that patients who survived sustained positive ratings across physical, emotional, functional, and social dimensions, thus underscoring the efficacy of the regimen beyond mere survival.

Distant metastasis represented the primary mode of treatment failure, thereby highlighting the need for improved systemic control. Several factors may contribute to this scenario, including ICI-induced immune activation being potentially dampened by lymphopenia during concurrent CRT, as previous studies have linked lymphocyte depletion to diminished immunotherapy efficacy.^34^ Additionally, patients with advanced ­disease and a heavy tumor burden may already harbor micrometastases prior to treatment initiation. Whether the intensity and schedule of current immunotherapy regimens are sufficient for systemic control remains unknown. Future studies should focus on (1) optimizing immunotherapy sequencing, (2) developing combinatorial strategies, and (3) implementing risk-adapted approaches to augment systemic control. Furthermore, this study has several limitations. First, it is a single-center, single-arm, exploratory phase II trial lacking a randomized control group, thus potentially introducing selection bias. Second, the sample size was relatively small, thereby limiting the statistical power. Consequently, exploratory subgroup analyses (eg, according to response status, PD-L1 expression, or lymphopenia grade) are underpowered and should be interpreted as hypothesis-generating. Despite a median follow-up of 25.5 months, long-term outcomes and late-stage toxicities necessitate continued monitoring. Additionally, the QoL data were obtained solely from surviving patients, which may have led to an overestimation of the overall benefit. Consequently, our findings should be validated in large-scale, multicentre, randomized controlled trials.

In conclusion, this study demonstrated that the combination of induction chemoimmunotherapy followed by sequential concurrent chemoradiotherapy and immunotherapy represents a viable and efficacious approach for managing local ESCC. This treatment regimen yields promising survival rates, tolerable adverse effects, and positive effects on QoL, even in high-risk patient populations. Given that distant metastasis remains the primary cause of treatment failure, future investigations should prioritize enhancing systemic disease control.

## Data Availability

The data sets and materials in this study are available from the corresponding author.
